# Agreement Between Proxy-Rated and Self-Reported Quality of Life for Persons With Dementia

**DOI:** 10.1093/geroni/igaa057.840

**Published:** 2020-12-16

**Authors:** Mariko Sakka, Ayumi Igarashi, Chie Fukui, Maiko Noguchi-Watanabe, Asa Inagaki, Manami Takaoka, Hiromi Kobayashi, Noriko Yamamoto-Mitani

**Affiliations:** The University of Tokyo, Tokyo, Japan

## Abstract

While quality of life (QOL) is an important endpoint of homecare for persons with dementia (PWD), PWDs often have difficulty in articulating their QOL by themselves. Instead proxy-rating is often used. However, evidence is still scarce regarding to what extent proxy-ratings reflect actual QOL of PWDs. We examined the association between self-report QOL by PWDs and proxy-rated QOL. We conducted a questionnaire survey to PWDs who were 75 years and older, their family, and homecare nurse in charge of the PWD. Two measures were used: 1) a newly developed, 4-item self-report for QOL of PWDs, and 2) a standardized, 6-item proxy-rating dementia QOL scale. In the self-report, the PWD were asked about their daily mood or satisfaction in life in brief, easy-to-understand sentences. The self-reports and proxy-ratings were compared using intraclass correlation coefficients (ICC). Data from 382 PWDs, 248 family caregivers and 124 nurses were used. The mean age of PWD was 85.9 years and 60.5% were female. The proxy-rating by nurses were more strongly associated with self-reports, compared to the association between family proxy rating and self-reports (r = 0.351, p < .001; r = 0.236, p < .001, respectively). Proxy ratings by spouses and biological children were significantly associated with self-report (r = 0.257, p =.004; r =. 204, p = .006, respectively), while rating by children-in-law were not (r = 0.217, p = .160). Proxy-ratings may not be an appropriate substitute for self-report. Homecare nurses may evaluate the QOL of PWD better than their family caregiver.

